# Pleomorphism in Soft Tissue Sarcomas: Molecular Characteristics and Clinical Features

**DOI:** 10.3390/medsci14050585

**Published:** 2026-09-18

**Authors:** Elena E. Kopantseva, Alexey S. Rzhevskiy, Ekaterina A. Lesovaya, Vlada E. Tuliavko, Nikolay A. Kozlov, Timur I. Fetisov, Kirill I. Kirsanov, Evgeny V. Denisov, Marianna G. Yakubovskaya

**Affiliations:** 1 Research Institute of Molecular and Cellular Medicine, Peoples’ Friendship University of Russia (RUDN University), Moscow 115093, Russia; rzhevskiy-as@rudn.ru (A.S.R.); lesovaya-ea@rudn.ru (E.A.L.); vlada.tuliavko@gmail.com (V.E.T.); n.kozlov@ronc.ru (N.A.K.); t.fetisov@ronc.ru (T.I.F.); k.kirsanov@ronc.ru (K.I.K.); 2 Institute of Molecular Theranostics, Sechenov First Moscow State Medical University, Moscow 119991, Russia; 3 Oncology Department, Ryazan State Medical University, Ryazan 390026, Russia; 4 N.N. Blokhin National Medical Research Center of Oncology, Moscow 115478, Russia; 5 Cancer Research Institute, Tomsk National Research Medical Center, Russian Academy of Sciences, Tomsk 634009, Russia

**Keywords:** pleomorphism, soft tissue sarcoma, genomics, transcriptomics, therapeutic advances

## Abstract

Pleomorphism in cells encompasses a variety of morphological features, such as heterogeneity in the size and shape of cells and nuclei, irregularity of the nuclear membrane, hyperchromasia, and peculiarities of cell organelles. Pleomorphism in mammalian cells is associated with genetically unstable neoplasms and is frequently accompanied by high rates of proliferation and low differentiation status. Among soft tissue sarcomas, a group of tumors characterized by the pleomorphic phenotype can be distinguished. Pleomorphic soft tissue sarcomas are a rare, understudied group of soft tissue sarcomas with aggressive behavior, low survival rates, and high resistance to standard chemotherapy treatment. Knowledge of the mechanisms behind the pleomorphic features, common genetic and epigenetic alterations, and their association with aggressive tumorigenesis is essential for advancing patient care for this group of sarcomas. In this narrative review, we consolidate existing evidence concerning clinical features of pleomorphic sarcomas, their possible histogenesis, the common drivers and transcriptional networks, and the potentially available approaches for treatment.

## 1. Introduction

Pleomorphism in cytology refers to the morphological heterogeneity between individual cells in a given population. The description of this phenomenon includes variations in cell size and shape, peculiarities of nuclei including shape, structure, membrane irregularities, hyperchromasia and location, and ratio and spatial distribution of cellular organelles [1,2]. In some strains of bacteria, yeast, and mycoplasma, this phenomenon is a part of the adaptation to environmental cues [3,4,5,6]. However, in mammalian cells, pleomorphism, especially nuclear pleomorphism, is commonly associated with genomic instability and neoplastic growths [7,8,9]. The pleomorphic component is observed in a wide range of neoplasms, from epithelial to mesenchymal and from benign to fully malignant tumors. Pleomorphic cancers demonstrate aggressive characteristics and poor prognosis, in comparison with their non-pleomorphic counterparts. For example, pleomorphic rhabdomyosarcoma (pRMS) has a worse prognosis and survival than other rhabdomyosarcoma (RMS) subtypes [10]. Many pleomorphic neoplasms are rare and understudied, which results in a lack of therapy options for patients with this diagnosis.

Pleomorphic soft tissue sarcomas (STS) are rare malignant neoplasms originating from mesenchymal cells and united by high morphological heterogeneity [1]. This list includes several subtypes of STS that are defined as pleomorphic entities according to the latest classification from the World Health Organization (WHO): undifferentiated pleomorphic sarcoma (UPS), pRMS, pleomorphic liposarcoma (pLPS), and myxoid pleomorphic liposarcoma (mpLPS) [11]. Additionally, several other subtypes of STS are frequently included in the discussion of pleomorphism [1] due to the pleomorphic component they display. Myxofibrosarcoma (MFS), previously grouped together with UPS under the umbrella term of malignant fibrous histiocytoma (MFH), is a myofibroblastic tumor [11] with a significant pleomorphic component [12]. Dedifferentiated liposarcoma (DDLPS) displays pleomorphism as a part of its dedifferentiated component [13]. Poorly differentiated leiomyosarcoma (LMS) and anaplastic malignant peripheral nerve sheath tumor (MPNST) display marked pleomorphism and an almost UPS-like appearance [14,15]. For the purposes of this review, we use the umbrella term pleomorphic STS to refer to UPS, MFS, pRMS, pLPS, DDLPS, LMS, and MPNST.

The common clinical features of pleomorphic STS include high ability to mimic other tumors, aggressive rapid growth, plasticity and atypical growth patterns, high rates of local recurrences and distant metastasis, and chemoresistance [16,17,18,19,20]. Selective treatment of pleomorphic STS relies primarily on accurate histological and molecular verification of these neoplasms. The diversity of tissues giving rise to these tumors results in a highly heterogeneous cellular structure, making definitive histogenesis and diagnostics of pleomorphic STS subtypes one of the most complicated areas in cancer pathology.

The molecular characteristics of pleomorphic STS leave many unresolved questions at present. Data on the genomic drivers and transcriptional programs are available for the most common pleomorphic subtypes. However, even for these subtypes, an examination of larger cohorts of patients may lead to the discovery of new molecular alterations. Most importantly, the molecular mechanism behind the emergence, evolution, and functioning of the pleomorphic tumor component in STS has not been elucidated at present. New data are required to fill in these gaps, while careful examination of the available studies can help identify common links and processes between the pleomorphic STS subtypes.

This review brings together the currently available knowledge on pleomorphic STS as a group. We outline the documented clinical features of each pleomorphic STS subtype and analyze the common genetic drivers, transcriptional and epigenetic traits, cells of origin, and available therapeutic options. Finally, we summarize open challenges and directions for future studies for this area of sarcoma research.

## 2. Review Methodology

This narrative review was informed by searching the databases Pubmed (MEDLINE), Google Scholar, Cochrane, and ClinicalTrials.gov, and it covered the time period from 1994 to 2026. To find an optimal amount of information about all pleomorphic STS, even the rare subtypes, we searched for the following terms: “undifferentiated pleomorphic sarcoma”, “myxofibrosarcoma”, “pleomorphic liposarcoma”, “dedifferentiated liposarcoma”, “pleomorphic rhabdomyosarcoma”, “leiomyosarcoma”, and “malignant peripheral nerve sheath tumor”. These primary search terms were also combined with the following terms: “genetics”, “transcriptomics”, “clinical trials”, “clinical features”, “cell signaling”, and “morphology”. The acquired results were filtered to include articles with full text available in English. Reviews and full-length experimental studies were prioritized. Additionally, articles published after 2000 were prioritized, since the classification of STS is constantly evolving. For example, until the early 2000s, the outdated term MFH was frequently used for UPS and MFS.

## 3. Clinical Traits of Pleomorphic STS

The pleomorphic STS group encompasses the following histotypes: UPS, MFS, pRMS, pLPS, DDLPS, LMS, and MPNST [11]. Additionally, the mpLPS and pleomorphic dermal sarcoma (PDS) also display pleomorphic traits [21,22], but due to their extreme rarity and insufficient data, they are not analyzed in this review. The main histopathological features of the pleomorphic STS subtypes are summarized in Table 1.

### 3.1. Undifferentiated Pleomorphic Sarcoma

UPS is defined by WHO as the malignant tumor of uncertain differentiation [11]. It constitutes up to 15–20% of all STS cases [39,40], manifests mostly in the extremities and trunk, and predominantly affects patients over 40 years of age [23]. Its frequency of local recurrences is 20–30%, and the frequency of metastases is 30–35% [24,25]. The most common metastases locations include lungs, bone, and liver [24,41]. The 5-year overall survival rate is 50–60% [25,42], with the prognostic factors including tumor size, histological grade, and presence of distant metastases during the primary diagnosis [24,25]. Due to its definition, UPS is considered to be a “wastebasket” of different undifferentiated sarcomas, and therefore is also the most heterogeneous STS type [43]. Another unique trait of UPS is that it constitutes the majority (42%) of radio-associated sarcomas, even capable of being induced by therapeutic radiotherapy [44,45]. It is also the most common sarcoma subtype to occur in patients with a predisposition to tumorigenesis, including Li–Fraumeni syndrome [46].

### 3.2. Myxofibrosarcoma

MFS is a malignant myofibroblastic tumor [11] with the marked presence of myxoid and pleomorphic components [12,43]. It manifests as a slow-growing subcutaneous tumor mass in extremities and adjacent areas, and is one of the most frequent sarcomas in elderly patients, with its occurrence peaking in the sixth to eighth decade of life [26]. The overall 5-year survival rate is 70% [47]. The characteristic trait of MFS is the longitudinal extension of malignant cells along the fascial planes, which is detected by magnetic resonance imaging (MRI) as peritumoral infiltration outside of visible tumor boundaries (“tail-like growth”) [19]. This quality presents a challenge for surgical excision and leads to the high frequency of MFS recurrences (50–60%). The metastatic potential is reliably predicted by the histological grade: low-grade MFS almost never metastasizes, while mid-grade and high-grade MFS with a higher degree of pleomorphism may result in a 16–38% metastasis rate [12]. In addition to classical clinical predictors (tumor size, histological grade, and resection margins status), the percentage of the myxoid component in MFS correlates with survival rate: the presence of more than 5% of the myxoid component is associated with a better outcome [48].

### 3.3. Pleomorphic Rhabdomyosarcoma

PRMS is the pleomorphic subtype of the malignant skeletal muscle tumor, RMS [11]. It is the most frequent RMS subtype in elderly patients [11]. The tumor occurs predominantly in the lower extremities of elderly male patients in the sixth to seventh decade of life [27], as confirmed by a recent population study on the Japanese collection of soft tissue and bone tumors [28]. The prognosis for this tumor type is one of the worst out of all the pleomorphic STS. The frequency of local recurrences is 53.8%, and more than 80% of the tumors metastasize over the course of five years. The median survival rate for localized tumors is 12.8 months and for metastatic tumors it is only 7.1 months, with the 2-year and 5-year pRMS survival rates suggested to be 66.3% and 54.1%, respectively [28]. Large tumor size, old age, and distant metastases are associated with worse prognosis [28,49]. Studies suggest a strong similarity between pRMS and UPS [50], perhaps due to RMS and UPS having overlapping cells of origin [51]. Like UPS, pRMS sometimes arises as a result of radiation treatment [52,53], and there have been cases of pRMS in pediatric patients associated with the Li–Fraumeni syndrome [54].

### 3.4. Pleomorphic Liposarcoma

PLPS is the rarest, pleomorphic subtype of the malignant adipogenic tumor, liposarcoma (LPS) [11,55]. It is the most aggressive subtype of LPS [56], with local recurrence and metastasis rates of higher than 30%, and a 5-year overall survival rate of 60% [29]. PLPS mainly targets elderly patients with incidence rates peaking around the sixth decade of life, with slight prevalence in male patients [29]. Its predominant location is in the deep soft tissues of limbs, especially the hip and lumbo-pelvic area, with the notable exception being the retroperitoneal area [1]. Adverse prognostic factors include localization in places other than extremities, large tumor size, and high mitotic index [29]. Several studies have described cases of pLPS having an additional epithelioid differentiation or myxoid component [29,57,58], demonstrating high plasticity of this sarcoma subtype. Modern studies also emphasize this subtype’s ability to mimic other LPS subtypes and the need for its fast and correct diagnosis, considering its high rate of local recurrences and metastases [59].

### 3.5. Dedifferentiated Liposarcoma

DDLPS is an LPS subtype that develops from the well-differentiated liposarcoma (WDLPS) [60]. It predominantly affects patients over 50, with its key localization being in the retroperitoneum, followed by the extremities, trunk, scrotum, and spermatic cord [30]. DDLPS displays lower metastatic potential (19%) than other pleomorphic sarcomas but is associated with a higher recurrence rate (40–80%), especially in the retroperitoneum [31]. The disease-related mortality rate is 42% [61], with the histological grade of the dedifferentiated component correlating with the survival rate [62]. Interestingly, in rare cases, DDLPS displays the presence of myogenic, rhabdomyoblastic, or osteoblastic differentiation [62,63,64]. Reports vary on how this line of differentiation impacts clinical prognosis. Myogenic and rhabdomyoblastic DDLPS have demonstrated worse survival statistics than regular DDLPS [62], but there are also reports of a prognostically favorable DDLPS subtype with rhabdomyogenic differentiation [65].

### 3.6. Leiomyosarcoma

Leiomyosarcoma is classified by WHO as a malignant smooth muscle tumor [11], and it is considered as one of the more common STS types [66]. Depending on the location, the reported rate of local recurrence is 10–43% [33], metastasis rate is 40–89%, mortality rate is 50–65%, and median survival rate is 9–17 months. The most frequent localization of LMS metastases is the lungs [34]. Less than 10% of LMS may be poorly differentiated with explicit pleomorphism, but usually some parts with classical LMS histology are still present [35]. The loss of myogenic markers in LMS has been reported to have adverse prognostic value [14]. However, there is also data that poorly differentiated LMS with some myogenic gene expression might have higher metastatic potential than the undifferentiated pleomorphic sarcoma (UPS), but lower potential than LMS [67]. On the molecular level, these pleomorphic LMS are close to or indistinguishable from UPS, so the term “pleomorphic sarcomas with partial myogenic phenotype” did not receive wide distribution in clinical practice [67].

### 3.7. Malignant Peripheral Nerve Sheath Tumor

MPNST is a rare STS type (3–10%) that originates from the peripheral nerves or displays differentiation of components of the nerve sheath, and it was known formerly as the malignant schwannoma [68,69,70]. The average 5-year survival rate is 30–60% [71,72], with around 40% of patients developing distant metastases within 5 years and around 30% of patients having local recurrence within 10 years [37,38]. The median onset is 30–60 years, which is slightly younger than for other genomically complex sarcomas [36]. The main predisposing factor to MPNST is the presence of neurofibromatosis type I (NF1), with NF1 patients being 1000–2000 more likely to develop MPNST than the non-NF1 population [73]. Around 10% of MPNST can occur as a result of radiation therapy for a different tumor, but these cases are more rare than for UPS [74]. The presence of NF1 association and induction through radiation are considered to be major negative prognostic factors [75,76]. Pleomorphic features are frequently reported for MPNST [77,78,79], although, similarly to LMS, there appears to be a morphological spectrum ranging from cases with distinguishable neurogenic differentiation to pleomorphic cases [15].

### 3.8. Chemoresistance in Pleomorphic STS

The question of chemoresistance is critically important for understanding the clinical dynamics of pleomorphic STS. Patients with advanced or metastatic pleomorphic STS face a dismal prognosis, and chemotherapy regimens, including doxorubicin as a monotherapy and in combination with other drugs, remain the standard for treatment [80]. This indicates the lack of alternatives for the patients who are unresponsive to anthracyclines. Molecular studies emphasize that pleomorphic sarcomas have a complex genetic background with inactivation of common tumor suppressors, but they are missing the identified initiating oncogenic events, which could serve as targets for therapy [1]. Present studies confirm that pRMS may be less chemosensitive than other pleomorphic STS subtypes: neoadjuvant/adjuvant chemotherapy does not improve overall, metastasis-free, or local recurrence-free survivals [20,81]. In the future, molecular analysis may provide theranostic and important prognostic information.

## 4. Genomic Drivers

**Figure 1:**
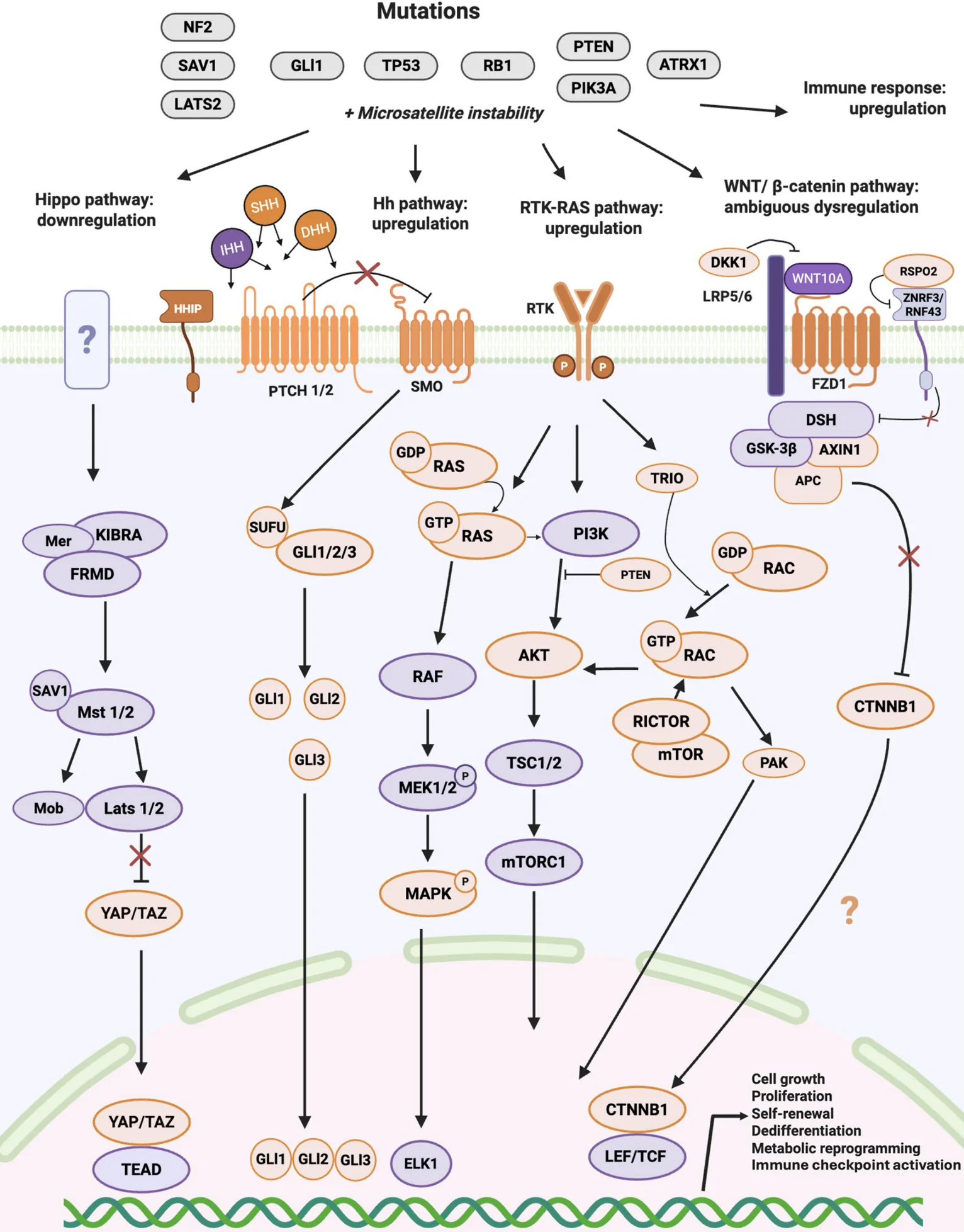
Schematic overview of the common mutations and transcriptomic changes in various pleomorphic STS subtypes. The common mutations in pleomorphic STS subtypes (shown in gray) lead to diverse downstream effects. The Hippo pathway is often negatively dysregulated due to NF2 (Merlin), SAV1, and LATS2 inactivation (shown in purple), which leads to the YAP/TAZ complex stabilization and relocation to the nucleus (shown in orange). Conversely, Hedgehog and RTK-RAS pathways are frequently activated due to upregulation of their components SHH/DHH, HHIP, PTCH1/2, SMO, SUFU, GLI1/2/3 (Hedgehog) and RTKs (AXL), RAS, RAC, TRIO-RICTOR-mTOR-AKT, and PAK (RTK-RAS) (shown in orange). An activator of the Wnt/β-catenin pathway (RSPO2) and its inhibitor (DKK1) are upregulated in some pleomorphic STS (shown in orange). Upregulation of Wnt/β-catenin pathway components (APC, AXIN1, CTNNB1) is also observed in some pleomorphic STS (shown in orange). The signaling network enables a transcriptional program that leads to increased proliferation, dedifferentiation, metabolic reprogramming and immune checkpoint activation.

Pleomorphism in STS cytology mostly arises from genetic instability, followed by multiple genetic abnormalities, including gene mutations, insertions and deletions, chromosome translocations, and DNA copy number variations [1]. Complex genetic features of pleomorphic STS are difficult to classify, but several similarities can be highlighted (Figure 1).

### 4.1. TP53/RB1

The key drivers of genetic instability present the abnormalities associated with DNA damage and repair signaling as well as apoptotic pathways. Indeed, the mutations in *TP53*, encoding the main regulator of genome integrity and apoptosis induction, as well as related signaling, are reported to be found in pleomorphic STS. More specifically, mutations in *TP53* are not usually high in LPS, but for pleomorphic LPS and DDLPS, *TP53* mutations are detected in 60% of cases [82,83,84]. This is tightly associated with *MDM2* amplification, leading to functional inactivation of non-mutated *TP53* [85,86,87,88]. The abnormalities in another tumor suppressor gene, *RB1*, are also reported for PLPS [83,88]. Additionally, DDLPS is characterized by the genetic abnormalities specific for chromosome 12 and affected, in addition to the *MDM2* gene, the *CDK4*, *HMGA*, *SAS*, and *GL1* genes [86]. Mutated *TP53* and oncogenic *KRAS* in pRMS are not only associated with progression and metastasis but also result in the development of the pleomorphic phenotype, which was supported by the study in vivo in adult mice [89,90,91,92]. PRMS with the complete loss of both *TP53* and *RB1* are associated with lower immune infiltrate than pRMS with higher levels of either of those proteins [93]. *TP53* mutations/deletions as well as loss of chromosome 13q, which leads to RB1 inactivation, occur in approximately 80% of pLMS and 70% of UPS [84,94,95,96,97,98]. Mutations in *TP53* and other genomic changes have been described in MPNST [99].

### 4.2. MSI

Other drivers of genome instability (GIN) and high tumor mutation burden (TMB) are the disruptions in the mismatch repair system. Microsatellite instability (MSI) is frequent in colorectal cancers (CRC). However, several clinical cases of patients with a family history of MSI-driven colorectal cancer or with primary colorectal tumor/Lynch syndrome/Muir-Torre syndrome developing pRMS, pLMS, and UPS have been described [20,100,101,102,103,104,105]. The disruption of *MSH2* and *MSH6* genes was reported for UPS and pRMS associated with hereditary non-polyposis colorectal cancer [106]. In line with this observation, LMS development could be tightly associated with Li–Fraumeni syndrome, with germline *TP53* mutation as the key pathogenic driver, or with primary hereditary retinoblastoma with *RB1* loss [107,108,109,110].

### 4.3. ATRX/Telomeres

Alternative lengthening of telomeres and loss of tumor suppressor-encoding *ATRX* (α-thalassemia/mental retardation syndrome X-linked) gene expression is reported for UPS, pRMS, pLPS, DDLPS, and pLMS [111,112,113].

### 4.4. PIK3CA/AKT/mTOR Pathway

Chromosomal deletions encompassing *PTEN*, the gene encoding the suppressor of the PIK3CA/AKT/mTOR pathway, have been described for 40–50% of LMS and 20–25% of UPS [85]. For pRMS, the genomic alterations in the AKT pathway genes occur in 80% of the cases [81].

### 4.5. Hippo Pathway

According to CNV analysis of 108 sarcoma samples, 40% of LMS, DDLPS, MFS, UPS, and UPS with giant cells display copy number loss of the *NF2*, *SAV1*, and *LATS2* genes corresponding to the Hippo pathway [114]. This is a high percentage, considering that mutations in this pathway are uncommon, and only *NF2* is recognized as a bona fide tumor suppressor gene [115].

## 5. Transcriptomic Landscape of Pleomorphic Sarcoma

Genomic alterations in pleomorphic STS lead to a network of downstream transcriptomic changes (Figure 1). *TP53* is a crucial regulator of multiple networks, which include the Hippo, Hedgehog, Wnt, Notch, and PI3K-TOR pathways [116,117]. Its inactivation is known to alter the lipid and glucose metabolism, lysosome autophagy, and properties of the immune microenvironment [116,118]. Inactivation of *RB1* affects cell cycle genes and lipid metabolism [117,119]. Mutations in the *RAS*, *PTEN*, and *GLI* genes dysregulate the Ras, PI3K-TOR, and Hedgehog signaling pathways, respectively. In this section, we discuss transcriptional alterations in these pathways in pleomorphic STS and their possible role in maintaining the pleomorphic phenotype.

### 5.1. Hippo/YAP/TAZ Signaling

The Hippo pathway is a control system of cell proliferation and organ size. Negative dysregulation of the Hippo pathway leads to the translocation of YAP/TAZ proteins into the nucleus and the activation of genes related to proliferation, apoptosis, and stem-cell renewal [120].

Negative deregulation of the Hippo pathway and stabilization of YAP/TAZ transcription activation occur in UPS, UPS with giant cells, MFS, DDLPS, and LMS [114]. Pleomorphic sarcomas display activation of *FOXM1*, one of the downstream effectors of the YAP/TAZ transcriptional complex. Elevated expression of *FOXM1* is detected in UPS, LPS, and LMS, while in a non-pleomorphic, translocation-positive synovial sarcoma (SS), its expression is less uniform [114]. FOXM1 leads to activation of proliferation and induction of pluripotency genes (*SOX2*, *KLF4*, *OCT4*, and *NANOG*) in UPS and LPS [121]. In RAS-driven RMS (eRMS and pRMS), YAP is critical for tumor maintenance, as it supports tumor cell proliferation and inhibition of differentiation [122]. In the autochthonous mouse model of UPS, YAP promotes proliferation and dedifferentiation through suppression of *USP1* expression and subsequent activation of NF-κB signaling [123].

Based on the present data, there is some correlation between the pleomorphic phenotype in STS (UPS, LPS, LMS) and the negative dysregulation of the Hippo pathway, when compared to non-pleomorphic sarcomas (SS). The mouse model of UPS also provides a possible mechanism linking YAP/TAZ-mediated transcription to the maintenance of the pleomorphic state in pleomorphic STS. More evidence needs to be acquired for pLPS and poorly differentiated MPNST.

### 5.2. Hedgehog Signaling

Hedgehog signaling regulates the transcription of cell cycle components, thereby controlling development, tissue repair, stem cell homeostasis, and tumorigenesis [124]. It is implicated in CSC maintenance in multiple leukemias and solid cancers [125].

Components of Hedgehog (*GLI1*, *PTCH1*, and *HHIP)* and Notch signaling (*HES1*, *HEY1*, and *HEY2*) are upregulated in the stem-like tumor population in UPS, and the combined inhibition of these pathways leads to tumor size and transplantation potential reduction [126]. In pRMS, *PTCH1/2* and *SMO* are overexpressed [81]. Anaplastic eRMS, a pediatric equivalent of pRMS, displays elevated levels of *GLI1* compared to non-pleomorphic eRMS cases [127]. GLI2-mediated signaling is upregulated in the dedifferentiated component of DDLPS compared to WDLPS, and is considered to be linked to dedifferentiation, ECM remodeling, and an immune-poor tumor microenvironment (TME) [128]. Elevated expression of *SMO*, *SUFU*, and *GLI1* occurs in uterine LMS and leiomyomas with atypical and pleomorphic features [129]. NKX-6 upregulates the SHH pathway in LMS, and its overexpression promotes the stem-like program (*KLF8*, *MYC*, *CD49F*) in LMS tumor cells [130]. In MPNST, Hedgehog signaling components have variable levels of expression. *SHH* and *GLI1* are activated in NF1, while in the MPNST, *DHH* and *PTCH2* are downregulated [131]. It is proposed that MPNST has two subtypes, and the SHH signaling (the *SMO*, *GLI2*, *GLI3*, *CCNE1*, and *TGFB2* genes) is activated in one of them [132]. This MPNST-G1 subtype has poorer immune TME and worse prognosis and may originate from neural crest cells, which might suggest that it is the poorly differentiated, pleomorphic MPNST variant.

Based on these data, upregulation of Hedgehog signaling has correlative ties to the pleomorphic STS. It is preferentially activated in pleomorphic and poorly differentiated STS subtypes (stem-like tumor cells in UPS, anaplastic eRMS, DDLPS, poorly differentiated MPNST) compared to STS subtypes with higher differentiation (WDLPS, well differentiated MPNST), although more comparative studies are required.

### 5.3. Wnt Signaling

Wnt networks (Wnt/β-catenin, noncanonical Wnt signaling, and Wnt/Ca^2+^ pathways) are implicated in stem cell maintenance, proliferation, metabolic processes, and cell migration [133]. The Wnt/β-catenin pathway is crucial for the maintenance of cancer stem cells [134].

Activation of the Wnt/β-catenin pathway through uncomplexed β-catenin is detected in around 50% of STS across subtypes: UPS—60%; LMS—75%; and LPS—25% [135]. For MPNST, it has been proposed that there are two subtypes, with one of them having a better prognosis and activated Wnt/β-catenin signaling *(WNT10A*, *RAC2*, *AXIN1*, and *FZD1*) [132]. In pRMS, *CTNNB1* and *APC1* are upregulated [81]. Heterogeneous activation of the Wnt/β-catenin pathway among UPS cases is linked to their immune status. UPS with activated Wnt/β-catenin is immune-cold, while UPS with downregulated Wnt/β-catenin is immune-hot [136].

Expression of the R-spondins (the *RSPO* gene family), the Wnt/β-catenin pathway activators, is elevated in some pleomorphic STS. In pRMS, *RSPO2* expression is upregulated in one of the pleomorphic, non-myogenic tumor clusters [137]. In MPNST, *RSPO2* knockdown triggers a loss of viability in tumor cells [138].

DKK1, an inhibitor of Wnt/β-catenin signaling, is upregulated in a subgroup of UPS, which coincides with the immune-hot TME [136,137]. The DKK1^+^ UPS tumor cluster with stem-like properties was detected in the single-cell study [139]. *DKK1* is commonly upregulated in MPNST cell lines [140]. Furthermore, downregulation of the Wnt/β-catenin pathway in a sarcoma cell line leads to a decrease in proliferation but fails to override the differentiation block [135]. The inactivation of the Wnt pathway in hMSC cells leads to the formation of MFH (UPS-like) tumors [134].

Based on present data, the role of the Wnt/β-catenin pathway in the pleomorphic STS remains contradictory. There is an association of both inhibitors (UPS, MPNST) and activators (pRMS, MPNST) of this pathway with pleomorphic, stem-like tumor populations in STS. However, this pathway is involved in genesis of some non-pleomorphic sarcoma types, like SS [141,142], which indicated that the activation of this pathway is not pleomorphic subtype specific.

### 5.4. Notch Signaling

Notch signaling is a conserved network involving a system of Notch receptors, enzymes, and specialized ligands to perform functions related to cell differentiation, apoptosis, NF-kB activation and metastasis, angiogenesis, and proliferation [143].

In UPS, components of Notch signaling (*HES1, HEY1*, and *HEY2*) and the Hedgehog pathway are upregulated in the stem-like tumor population, which affects tumor growth and viability [126]. However, another UPS study identified a stem-like tumor population with elevated expression of *DLK1*, an inhibitory ligand of Notch signaling [139].

At present, the data on Notch signaling in the pleomorphic and non-pleomorphic STS are not sufficient to evaluate its role for pleomorphic STS as a united group.

### 5.5. RTK-Ras Signaling: PI3K-AKT-TOR and MAPK/ERK Pathway

Growth factor signaling through receptor tyrosine kinases (RTK) and the activation of the Ras GTPase lead to the activation of several pathways, including PI3K-TOR and MAPK signaling. The PI3K-AKT pathway regulates cell survival, metabolism, angiogenesis, and metastasis in cancer [144,145]. The MAPK/ERK (MAPK) pathway controls development, cell proliferation, differentiation, and death [145,146].

PI3K-AKT and RAS-MAPK pathways are activated in most UPS cases, with hyperactivation of RAS-MAPK correlating with UPS aggressiveness [136,147]. MFS has a tumor-specific mechanism by which integrin 10 initiates TRIO-RICTOR-mTOR-AKT signaling to drive tumorigenesis [43]. In pRMS, RTK/MAPK and AKT/PIK3CA/mTOR pathways are consistently upregulated [81,148], with studies on mice suggesting that the RAS-MAPK-ERK pathway may be important for disease initiation [90,92]. In pLPS and DDLPS, AKT and MAPK pathways are activated through the AXL RTK, with the activated expression of *AXL* being linked to increased migratory and invasive tumor characteristics [149]. In LMS, the well-differentiated subtype may display preferential loss of *PTEN* expression and overexpression of *RICTOR* [150], although there are also reports of all LMS subtypes having this feature [85]. In MPNST, MAPK signaling is frequently activated overall [151].

RTK-RAS signaling plays a substantial role in pleomorphic STS (UPS, MFS, pRMS, pLPS, DDLPS, LMS, MPNST) growth and progression, although the link to pleomorphism is unclear. Additionally, a subset of mLPS displays activation of AKT and inactivation of PTEN [152,153], and aggressive SS shows activation of AKT, mTOR, 4E-BP1, and CyclinD1 [154]. These data should be taken into account when considering the impact of RTK-RAS signaling on pleomorphic and non-pleomorphic STS.

### 5.6. Glucose and Lipid Metabolic Transcriptional Programs

Glucose and lipid metabolism dysregulation are common hallmarks of cancer progression. An upregulation in the glycolysis rate and mitochondrial biogenesis in pleomorphic STS (UPS, MFS, LMS, pLPS, and DDLPS but not mLPS) is associated with severe downregulation in gluconeogenetic enzyme FBP2 [155]. PLPS, and in particular pleomorphic areas, are marked by the elevated expression of SETD5, a glycolysis regulator of cancer stem-like cells [156]. A subset of UPS displays an upregulation of succinate dehydrogenase subunits (*SDHB*, *SDHC*, and *SDHD*), which leads to impaired SDH complex activity and accumulation of oncometabolite succinate [157]. The TP53/RB1 mouse model of pleomorphic sarcoma displays an upregulation of ALDH1A3 and PGAM2 glycolytic enzymes [158].

Adipogenic genes and sometimes even signatures are associated with many pleomorphic STS. CD248, suggested to function as a pre-adipocyte glycoprotein [159], is upregulated in UPS, LMS, and RMS compared to STS without the pleomorphic component, such as SS [160]. The expression of the adipogenic receptor protein PPARy is shared by pLPS, mLPS, DDLPS, and UPS [161]. The murine model of pRMS is characterized by pleomorphic morphology, intermediate differentiation, and a strong expression of lipid transporter Lamp1 and adipogenic genes (*Pparg*, *Fabp4*, *Cd36*, *AdipoQ*, and *AdipoR1*) [162]. Single-cell transcriptomics of pRMS tumor cells reveals an upregulation of lipid transport genes (*LAMP1*, *SORT1*, *FLOT1*, *PGRMC2*) [137].

Based on the available data, some components of the glucose and lipid metabolic pathways are differentially expressed in the pleomorphic STS (UPS, MFS, LMS, pLPS, DDLPS), compared to non-pleomorphic STS (mLPS). However, there is no evidence to tie these pathways to pleomorphism at present.

### 5.7. Immune TME Transcriptional Programs

Pleomorphic sarcoma tumor cells frequently lead to a pronounced immune response. More than 50% of pleomorphic sarcomas exhibit elevated PD-L1 and PD-1 expression, increased T-cell infiltration, and abundant tumor-associated macrophages [94,163,164,165].

The presence of two immune groups has been documented for many pleomorphic sarcomas. For UPS and pRMS, the existence of immune-high tumors with pro-inflammatory TME and multiple immune checkpoint markers (UPS—LAG3, *TIGIT*, *TIMD4*, *IDO1*, and *PD-L1*; pRMS—*LAG3*, *IDO1*, *IFI30*, *EOMES*, and *HAVCR2*) and immune-low tumors with poorer immune TME have been described [93,136,137]. Immune-low UPS is additionally enriched in stemness markers, Wnt/β-catenin, FGFR2-PI3K/Akt, and MAPK pathways [136]. A divide into two immune groups has also been documented for DDLPS [166]. Compared to UPS, MFS has decreased overall immunogenicity, with lower MHC antigen presentation, neutrophil activation, and unbalanced mitochondrial activity [167]. LMS is usually not described as having enrichment in immune-pathway genes or checkpoint genes [168,169,170,171]. This corresponds with data on pLMS displaying a lower response to single-agent immunotherapy compared to UPS [170,172,173]. However, a recent proteomic study identified immune-cold and immune-hot (classical and dedifferentiated) LMS groups [174].

Pleomorphic STS, especially UPS and DDLPS, demonstrate high counts of CD68^+^ and CD163^+^ macrophages compared to translocation-induced sarcomas [175]. Macrophage infiltration in UPS is uneven, with CD68^+^ high spots having enrichment in the *FOLR2*, *IL4R*, *IL13RA1*, *PDCD1LG2*, and *IDO1* genes, glycolysis, inflammatory response, and mTORC1 signaling [176]. CD47/SIRPA-positive macrophages are detected in UPS, pRMS, and pLPS [175,176,177]. Cell fusions between UPS tumor clusters and macrophages are observed in UPS and MFS but not in pRMS [137,139,178]. Additionally, pRMS macrophages display the presence of the immunosuppressive molecules SIGLEC1, SIRPA, CSF1R, and HAVCR2 [137].

Based on the available data, pleomorphic STS have predominantly high immune infiltration, with a group of immune-low cases observed as a part of heterogeneity within the same subtype (UPS, pRMS, DDLPS, pLMS). This quality contrasts with non-pleomorphic, translocation-positive STS, like SS and mLPS, that are typically completely immune-cold [179,180]. The link between MSI and strong lymphatic activation and overexpression of immune checkpoint proteins has been previously described for CRC [181], with studies further suggesting that the degree of MSI plays a crucial role in determining immune infiltration [182]. We can hypothesize that a similar mechanism exists for pleomorphic STS, with MSI-high pleomorphic STS forming an immune-hot group with high expression of immune checkpoints, while the MSI-lower group is detected by the immune system to a lesser degree. However, further studies are required to confirm this link and elucidate the concrete mechanisms.

### 5.8. Alternative Splicing Deregulation

Splicing dysregulation is a common feature present in many STS. Expression of 25, 16, and 6 splicing factors is associated with survival in the DDLPS, LMS, and UPS, respectively. For UPS, these splicing factors were identified as hnRNPA0, KHDRBS2, HNRNPA3, RBFOX2, PTBP2, and ZRANB2 [183]. Single-cell sequencing of UPS and MFS identified a tumor cluster with upregulated splicing factor genes (*SNRNP70*, *RSRP1*, and *LUC7L*) [178]. A possibly similar tumor cluster with increased expression of nuclear RNA retention factor LENG8 was reported in a case of pRMS [137]. The long non-coding RNA NEAT1 is upregulated during UPS metastasis and presumably exerts its action through interaction with RNA splicing factors [184].

At present, data on splicing regulation in pleomorphic STS are scarce, so no conclusions for the group as a whole can be drawn. Additionally, splicing dysregulation does occur in non-pleomorphic STS as well [185,186], so if this process does have an effect on pleomorphism it likely has to do with the involvement of specific mRNA-editing and splicing factors.

## 6. Epigenetics

Classification of pleomorphic STS is rarely improved by methylation profiling [187]. Several abnormalities leading to epigenetic machinery disruption were reported for distinct pleomorphic STS subtypes, for example, somatic mutations in the components of the polycomb repressive complex 2 (PRC2) or the complete loss of trimethylated histone 3 lysine 27 (H3K27me3) in MPNST [188,189]. Interestingly, 90% of radiation therapy-associated MPNST are characterized by a loss of H3K27me3 expression [190].

## 7. Cells of Origin

In addition to the mutational landscape, described earlier in this review, cells of origin may play a role in establishing the pleomorphic phenotype of the STS. The discussion of this topic is still mainly composed of questions, rather than answers. One of the reasons for this is the methodologic difficulties, such as the rarity of pleomorphic tumor material, the need for single-cell analysis and in vitro and in vivo proof of the cell-of-origin theory. Additionally, pleomorphic STS exhibit amazing plasticity, potential dedifferentiation and transdifferentiation [18,191]. This further complicates the discussion of their origin, as the least differentiated cell population may not equate to the earliest cell state in this case. Single-cell results must be viewed with caution, distinguishing between cell phenotype, state, and cells-of-origin.

Currently, single-cell datasets and analysis of developmental trajectories are available only for UPS and DDLPS. For UPS, pseudotime with verification by mRNA velocity analysis supports the hypothesis that UPS originates from mesenchymal stem cells. Murine models were also used to study the possibly overlapping origins of UPS and RMS. The Ptch1, p53, and/or Rb1 conditional model showed that UPS is more likely to evolve from Pax-7-expressing satellite stem cells, while maturing myoblasts are more likely to serve as sources for eRMS [192]. However, a conflicting murine model study posited that eRMS can originate from satellite stem cells, while UPS have fibroblastic cells of origin [193]. Single-cell analysis of DDLPS suggested that a tumor population resembling multipotent progenitors of white adipose tissue are at the common origins of both WDLPS and DDLPS [194].

At present, the limited single-cell and murine model evidence acquired on UPS and DDLPS suggests that multipotent stem cells (mesenchymal stem cells, satellite stem cells, adipose stem cells) may be potential candidates for cells of origin for pleomorphic STS. However, this hypothesis needs to be tested on other pleomorphic STS subtypes.

## 8. Therapeutic Approaches

Analysis of therapeutic strategies shows that direct targeting of the pleomorphic phenotype does not exist at present. For some pleomorphic STS subtypes, targeting of the molecular aspects that lead to dedifferentiation or the increased immunogenicity associated with genomic instability may be possible. The therapeutic landscape for pleomorphic STS encompasses established clinical treatments, investigational approaches supported by clinical trials, and preclinical/emerging strategies.

### 8.1. Established Clinical Treatments

Surgical resection with negative margins remains the cornerstone of treatment for localized pleomorphic STS, as complete resection is the only potentially curative approach [195]. For retroperitoneal and anatomically complex tumors, achieving R0 resection is often challenging, and complex multivisceral resection may be required.

Chemotherapy, primarily doxorubicin in monotherapy or in combination with ifosfamide, remains a first-line treatment strategy for the majority of pleomorphic STS [80]. According to retrospective studies, the frequency of objective response to this regimen for UPS and pLPS does not exceed 15–25%, while the median survival without progression is 4–6 months [196]. The dedifferentiated component in DDLPS frequently demonstrates primary resistance to anthracyclines [197]. For MFS and UPS that show progression on anthracyclines, the combination of gemcitabine and docetaxel may be effective [198]. However, there is no proof of chemotherapy having selective effects on the pleomorphic component.

Radiotherapy plays an important role in the multidisciplinary management of pleomorphic STS, particularly in the neoadjuvant and adjuvant settings. For localized high-risk STS, neoadjuvant radiotherapy is frequently used to facilitate resection and improve local control. The SU2C-SARC032 trial demonstrated that the addition of pembrolizumab to preoperative radiotherapy significantly improved 2-year disease-free survival in patients with high-grade UPS and DDLPS of the extremity [199]. However, the efficacy of radiotherapy varies across histotypes. MFS demonstrates relatively higher radiosensitivity, while UPS and DDLPS show more variable responses [26]. It is important to note that pleomorphic STS can also arise as radiation-induced sarcomas (particularly UPS, pRMS, MPNST), typically developing 5–15 years after therapeutic irradiation, with a prolonged latency period of up to 25 years reported in some cases [44,45,52,74,200]. Radiation-induced UPS carries a poor prognosis due to its aggressive biological behavior, high local recurrence rates, and limited therapeutic options. Surgical excision with negative margins remains the primary treatment, while chemotherapy and re-irradiation have more limited roles depending on disease extent [201]. For recurrent or unresectable disease, radiotherapy may provide palliative benefit and, in select cases, durable local tumor control.

### 8.2. Investigational Approaches (Clinical Trials)

#### 8.2.1. MDM2/CDK4 Inhibitors

DDLPS, in almost 100% of cases, displays amplification of the *MDM2* gene and co-amplification of *CDK4* in the 12q13-15 region [197]. This molecular defect is present in both the well-differentiated and the dedifferentiated (pleomorphic) components. Inhibitors of MDM2 (milademetan, brigimadlin) and CDK4/6 (palbociclib, abemaciclib, ribociclib) are being evaluated as candidates for targeting the pleomorphic component. In early clinical studies, MDM2 inhibitor monotherapy led to disease stabilization in some patients with DDLPS, but objective responses were rare, and acquired resistance developed rapidly [202]. The phase II/III Brightline-1 trial (NCT05218499) is comparing brigimadlin with doxorubicin in patients with advanced DDLPS, representing a pivotal investigation of MDM2 inhibition in this setting. Combinations of MDM2 and CDK4/6 inhibitors (siremadlin + ribociclib) have demonstrated moderate efficacy [203]. *MDM2* and *CDK4* amplifications are not typical for UPS, MFS, and pRMS [153].

#### 8.2.2. Immunotherapy

The use of immune checkpoint inhibitors has proven effective for some pleomorphic STS (Table 2). As mentioned above, the link between a high degree of MSI and immune-hot TME with increased expression of immune checkpoints has been demonstrated for cancers, like CRC [181,182]. A similar mechanism between the degree of genomic instability, immune phenotype and sensitivity to ICI therapy is discussed for pleomorphic STS [204]. The degree of MSI and genomic instability may determine the split into immune-hot and immune-cold cases and determine the susceptibility to immunotherapy, even within one pleomorphic STS subtype.

According to the SARC028 study, the anti-PD-1 agent pembrolizumab led to an objective response in 23% of patients with metastatic UPS [205,206]. In the neoadjuvant SU2C-SARC032 study, addition of pembrolizumab to radiotherapy in patients with high-grade UPS improved 2-year recurrence-free survival from 52% to 67% [199]. Double blockade of CTLA-4 and PD-1 (nivolumab and ipilimumab) in UPS demonstrated a 16% response rate in the Alliance A091401 study [207]. Response to immunotherapy correlates with the presence of dense CD8+ T cell infiltrates and tertiary lymphoid structures in the pleomorphic component TME [208].

However, responses are highly histotype-dependent. In DDLPS, pembrolizumab monotherapy shows lower efficacy (ORR ~ 10%) [206]. For pRMS and pLPS, no significant response to immunotherapy has been documented. The ENVASARC trial (NCT04480502) evaluates envafolimab (a PD-L1 inhibitor) alone or in combination with ipilimumab in patients with advanced UPS or MFS who have progressed on chemotherapy [209]. This histotype-specific approach reflects the recognition that immunotherapy benefit is not uniform across pleomorphic STS. Although, some studies suggest that doxorubicin treatment might help boost the efficacy of ICI therapy for immune-cold STS [210]. Immune cell therapy (TCR-T, targeted at cancer testis antigens MAGE-A4 and NY-ESO-1) also benefits SS and myxoid LPS, but not pleomorphic histotypes, where expression of these antigens is low or absent [211].

**Table 2:** Selected clinical trials of immunotherapy in pleomorphic STS.

Histotype	Treatment	Trial Phase	ORR/ Primary Endpoint	Clinical Relevance	Reference
UPS	Pembrolizumab (SARC028)	Phase II	23%	Established option	[206]
UPS	Nivolumab + ipilimumab (A091401)	Phase II	16%	Investigational	[207]
UPS/DDLPS	Neoadjuvant pembrolizumab + RT (SU2C-SARC032)	Phase II	2-year DFS 67% vs. 52%	Practice- changing	[199]
UPS/MFS	Envafolimab ± ipilimumab (ENVASARC)	Phase II	Pending	Ongoing	[209]
DDLPS	Pembrolizumab (SARC028)	Phase II	~10%	Limited activity	[206]
UPS, MFS, DDLPS	Nivolumab ± ipilimumab (A091401)	Phase II	Histotype -dependent	Investigational	[207,212]

### 8.3. Preclinical and Emerging Strategies

Promising new directions that are still in preclinical or early clinical trial stages include modulation of the myeloid compartment (inhibitors of CSF1R, e.g., vimseltinib) in combination with PD-L1 inhibition [209], as well as inhibitors of nuclear export (selinexor) for DDLPS [197]. Recent fundamental studies showed that deletions and loss of function of *RB1* and *TP53* in UPS and MFS correlate with increased levels of the oncoprotein Skp2 [213], opening new therapeutic possibilities for these subtypes.

Single-cell studies have revealed the complexity of the tumor microenvironment and identified potential markers of chemoresistance, including the expression of *KLF4, ULK1*, *LUM,* and *GPNMB* in tumor cells associated with doxorubicin resistance [214]. Characterization of chemosensitive and chemoresistant tumor clusters at the single-cell level may guide future personalized therapeutic approaches.

The *TP53* mutations present in most pleomorphic sarcoma subtypes may open up new opportunities for their treatment, but so far this approach has not been thoroughly explored. ONYX-015, an oncolytic adenovirus therapy targeting missense p53, was used on advanced sarcoma patients, but partial response was observed only for one patient with MPNST [215]. PC14374 showed a positive effect on the murine p53-Y220C sarcoma model by reducing the mutant p53 levels and increasing the levels of WT p53 [216]. Inhibition of HSP90, a stabilizer of mutant p53 in tumor cells, has inhibited the growth of mutp53-positive mouse sarcoma cells [217]. Perhaps, the possibility of targeting mutant p53 in pleomorphic STS will be explored in the future.

In summary, specific targeting of the pleomorphic tumor component is absent at present. MDM2/CDK4 inhibitors are aimed at the molecular driver of the dedifferentiated component in DDLPS only. Immunotherapy is effective for UPS and possibly MFS, but with marked histotype-dependent heterogeneity. For most pleomorphic STS subtypes (pRMS, pLPS, high-grade MFS without MDM2 amplification), no effective targeted therapy strategies exist. At present, pleomorphic STS are mainly treated through surgery, chemotherapy, radiotherapy, and palliative approaches, with immunotherapy for a limited number of histotypes.

## 9. Challenges and Future Perspectives

At present, the amount of scientific data on the pleomorphic STS, especially the rarer subtypes, is far from sufficient. There is an overall clinical picture for all pleomorphic STS subtypes, although studies on more patients could provide for more accurate diagnostics and improve the estimations of prognosis and survival for each subtype. The bulk transcriptomic data for most pleomorphic STS subtypes are available, but there is not enough information to characterize the pleomorphic to non-pleomorphic continuum for LMS and MPNST. The epigenetics of pleomorphic STS is largely undocumented and needs further research. However, the most evident gap in knowledge for pleomorphic STS is the single-cell studies. Currently, single-cell transcriptomic and/or spatial datasets are available for UPS [139,178,218], MFS [178], and DDLPS [194,219,220]. For pRMS, the published scRNA-seq dataset is based on a single case [137]. For pLPS, pLMS, and MPNST, there is no data on single-cell populations, even at the transcriptomic level. Considering the heterogeneous nature of pleomorphic STS, single-cell genomic, transcriptomic, proteomic, and spatial omics data are crucial for figuring out the mechanics behind these tumors. Single-cell studies would likely provide an answer to the question of cells of origin for some of the rarer pleomorphic subtypes. This method could also be used to integrate data from all pleomorphic STS and describe the common features and functions of the pleomorphic component in all of them. Most importantly, if supplemented with in vitro experimental approaches for chemoresistance prognosis as well as patient-derived xenografts in vivo, single-cell studies would hopefully provide candidate genes and proteins for targeted therapy for pleomorphic STS subtypes.

## 10. Conclusions

This review summarizes the available data on clinical genomic, transcriptional, and epigenetic features of pleomorphic STS, their cells of origin, and available therapy options. At present, the most common denominator for pleomorphic STS subtypes appears to be the complex karyotype with *TP53* and *RB1* mutations and genomic instability. The dysregulation of Hippo, Hedgehog and RTK-RAS signaling and the presence of immune-hot and immune-cold groups within one subtype are also common traits, but need verification on some of the rarer pleomorphic STS. UPS remains the most well-studied pleomorphic STS, while the other subtypes are associated with much less data. These gaps in knowledge, especially at the single-cell level, prevent deeper understanding of the pleomorphic sarcoma cells, their role in the tumor architecture, progression, and therapy resistance. Acquiring data on these topics will hopefully lead to effective and safe strategies for treatment of pleomorphic STS in the future.

## Figures and Tables

**Table 1:** Characteristics of pleomorphic STS.

STS Subtype	Peak Age Range	Typical Location	Recurrence/Metastatic Risk	WHO Classification Status	Histopathological Features	Molecular Features
UPS	>40 years [23]	Trunk and extremities [23]	20–30%/ 30–35% [24,25]	Malignant tumor of uncertain differentiation, pleomorphic [11]	Hypercellular mesenchymal tumor presented by haphazard combination of various growth patterns (including patternless areas) of malignant cells with highly variable in shape and size hyperchromic nuclei, variable amount of cytoplasm (including bizarre forms), high mitotic activity, fibro-sclerotic matrix and necrotic areas. The tumor cells have no signs of specific tissue differentiation [1].	Mutations in *TP53*, *RB1*, *ATRX*, *MSH2*, *MSH6,* Hippo signaling genes, *PIK3CA/AKT/mTOR* signaling genes [see Section 4 for references]
MFS	60–80 years [26]	Extremities and adjacent areas [26]	50–60%/ 0–38% [12]	Malignant fibroblastic/myofibroblastic tumor [11]	Cytologically analogous to UPS, producing a discernible myxoid matrix. Ability to produce myxoid matrix by tumor cells inversely correlates with histological grade [1,12].	Mutations in *TP53*, *RB1*, *ATRX*, Hippo signaling genes [see Section 4 for references]
pRMS	>60 years [27]	Lower extremities [27]	53.8%/ 80% [28]	Malignant smooth muscle tumor, pleomorphic subtype [11]	Cytologically analogous to UPS, with scattered cells or small groups of cells with histological signs of skeletal muscle differentiation [1].	Mutations in *TP53*, *RB1, ATRX, MSH2, MSH6, PIK3CA/AKT/mTOR* signaling genes [see Section 4 for references]
pLPS	60–70 years [29]	Limbs, hip and lumbar-pelvic area [1]	34%/ 32% [29]	Malignant adipocytic tumor, pleomorphic subtype [11]	Cytologically analogous to UPS, with scattered or small groups of cells with histological signs of lipoblast differentiation, including bizarre forms [1].	Mutations in *TP53* [see Section 4 for references]
DDLPS	>50 years [30]	Retroperitoneum [30]	40–80%/ 19% [31]	Malignant adipocytic tumor, dedifferentiated subtype [11]	Cytologically analogous to UPS, with prominent, even macroscopically, areas of the well-differentiated component with lipocytic differentiation [1].	Mutations in *TP53*, *RB1*, *DM2*, *CDK4*, *HMGA*, *SAS*, and *GL1* genes [see Section 4 for references]
LMS	40–50 years [32]	Extremities, retroperitoneum, uterus [33]	10–43%/40–89% [33,34]	Malignant smooth muscle tumor [11]	Poorly differentiated cases are cytologically analogous to UPS, with scattered cells or small groups of cells with histological signs of smooth muscle differentiation [1,35].	Mutations in *TP53*, *RB1* inactivation [see Section 4 for references]
MPNST	20–60 years [36]	Extremities, trunk, head and neck [36]	30%/ 40% [37,38]	Malignant peripheral nerve sheath tumor [11]	Poorly differentiated cases are cytologically analogous to UPS, without histological signs of specific tissue differentiation, originating from main nerve trunks [1,15].	Mutations in *TP53* [see Section 4 for references]

## Data Availability

No new data were created or analyzed in this study. Data sharing is not applicable to this article.
